# Exploring PTSD in emergency operators of a major University Hospital in Italy: a preliminary report on the role of gender, age, and education

**DOI:** 10.1186/s12991-018-0184-4

**Published:** 2018-05-04

**Authors:** Claudia Carmassi, Camilla Gesi, Martina Corsi, Ivan M. Cremone, Carlo A. Bertelloni, Enrico Massimetti, Maria Cristina Olivieri, Ciro Conversano, Massimo Santini, Liliana Dell’Osso

**Affiliations:** 1 Section of Psychiatry, Department of Clinical and Experimental Medicine, University of Pisa, Via Roma 67, 56100 Pisa, Italy; 2 Emergency Medicine and Emergency Room Unit, AOUP, Pisa, Italy

**Keywords:** Post-traumatic stress disorder (PTSD), Emergency personnel, Nurses, Health care assistants, Work and social functioning/adjustment, Age, Gender, Education

## Abstract

**Background:**

Emergency services personnel face frequent exposure to potentially traumatic events, with the potential for chronic symptomatic distress. The DSM-5 recently recognized a particular risk for post-traumatic stress disorder (PTSD) among first responders (criterion A4) but data are still scarce on prevalence rates and correlates.

**Objective:**

The aim of the present study was to explore the possible role of age, gender, and education training in a sample of emergency personnel diagnosed with DSM-5 PTSD.

**Methods:**

The Trauma and Loss Spectrum-Self-Report (TALS-SR) and the Work and Social Adjustment Scale (WSAS) were administered to 42 between nurses and health care assistants, employed at the emergency room of a major University Hospital (Pisa) in Italy.

**Results:**

21.4% of the sample reported DSM-5 PTSD with significantly higher scores in the TALS-SR domain exploring the acute reaction to trauma and losses among health care assistants, older, and non-graduated subjects. A significant correlation between the number of the TALS-SR symptoms endorsed, corresponding to DSM-5 PTSD diagnostic criteria emerged in health care assistants.

**Conclusions:**

Despite further studies are needed in larger samples, our data suggest a high risk for PTSD and post-traumatic stress spectrum symptoms in nurses and health care workers operating in an emergency department, particularly among health care assistants, women, older, and non-graduated operators.

## Background

The DSM-5 encoded important changes for what concern post-traumatic stress conditions, particularly post-traumatic stress disorder (PTSD). Besides changes in the symptomatological diagnostic criteria, the actual edition of the manual better specified Criterion A about the trauma eliminating the need of person’s response to the event involving *intense fear, helplessness, or horror* (criterion A2) and better clarifying the characteristics of the potentially traumatic experiences including, for the first time, *a repeated or extreme indirect exposure to aversive details of the event(s), usually in the course of professional duties (e.g., first responders, collecting body parts; professionals repeatedly exposed to details of child abuse)* (criterion A4).

Psychological distress in health care workers may vary across different specialties but increasing evidence highlights that staff operating in emergency planning, such as doctors, nurses, and paramedics, to be at high risk for PTSD [1]. Emergency departments, in fact, may be challenging because of frequent unpredictability of daily work cases, coping with the acute phase of most disorders, including traumatic incident exposure, frequently facing patients’ and their families’ expectations in unexpected and acute critical cases/situations. This induced researchers to investigate work-related psychological disorders, such as Burnout Syndrome, an occupational health concept of emotional and physical exhaustion, depersonalization and decreased personal accomplishment [2] and, most recently, PTSD [3, 4].

The prevalence of PTSD in the general population has been reported to be approximately 6.8% [5] with lower rates in Italy [6, 7], but studies on emergency services personnel have reported higher rates showing an increased risk in such populations. PTSD prevalence rates ranging between 10 and 17% have in fact been reported among emergency unit operators [4, 8–10], with even higher percentages (18–44%) being reported among nurses [3, 11]. In a study investigating the relationship between exposure to critical incidents and mental health problems among emergency medical care personnel, Ward et al. [12] found that symptoms of anxiety, depression, or PTSD intensified when exposure to critical incidents increased. However, the rate at which symptoms increased eventually slowed over time, suggesting that there may be a time-dependent desensitization to the effects of repeated work-related traumatic exposures [12]. Mealer et al. [3] investigated 332 University hospital nurses operating in the University of Colorado Hospital (USA), reporting a PTSD diagnosis in 18% of the sample, with 22% of it showing PTSD symptoms. An extensive systematic review of all empirical articles regarding emergency medical responders and conceptual literature on the constructs of interest in other related populations, reported exposure to traumatic events to be between 80 and 100%, and rates of PTSD higher than 20%. In this same review, a modification of the stress process model is suggested to explain the relationships among occupational stress exposure, PTSD, and high risk of alcohol and other drug use [13]. More recently, Fjeldheim et al. [14] reported 94% of 131 South African university paramedic trainees to have directly experienced trauma, with 16% meeting PTSD criteria and 7% chronic perceived stress, suggesting the need for efficient, ongoing screening of PTSD symptomatology in trauma-exposed high-risk groups. Emergency care workers trainees, in fact, have been suggested to be at an even higher risk of developing PTSD due to exposure to a novel environment, age, inexperience in the field, and the added pressure of academic evaluation [15]. Resilient coping strategies with mitigation of common maladaptive psychological symptoms may be in fact developed during the course of professional career as seen in some nurses [11].

Little data are yet available on DSM-5 PTSD rates among operators of the emergency units in Europe, particularly in Italy [16, 17]. Slight differences in PTSD symptomatological rates have been reported on subjects exposed to mass trauma and assessed according to either the DSM-IV or the DSM-5 PTSD criteria, suggesting the need for further investigation on the possible rates detected by means of the new criteria. The aim of the present study was to assess PTSD symptoms among nurses and health care working in the Emergency Unit of a major University Hospital in Italy.

## Methods

### Study design

A total sample of 42 subjects employed, at the time of enrollment, at the emergency room of the Azienda Ospedaliero-Universitaria Pisana (AOUP) was included in this study. According to the study protocol, the whole medical staff employed at the emergency room of the AOUP was asked to participate in the study with the only exception of the doctors. According to the Italian health care system, we thus included nurses and health care assistants. Medical doctors were excluded as, upon the organization of the services of the AOUP, they rotate between two different services, that are the emergency room and the emergency medicine, aim of the present study was to assess post-traumatic stress symptoms in the former department in order to have a more selected sample operating the first health care interventions.

The subjects were asked to fulfill the self-report instruments immediately before or after their work schedule.

The study was conducted in accordance to Helsinki Declaration and received the approval of the Ethics Committee of Area Vasta Nord Ovest Toscana.

### Instruments and assessment

All subjects were assessed by means of the Trauma and Loss Spectrum-Self-Report (TALS-SR) [18, 19], for post-traumatic stress spectrum symptoms developed after exposure to trauma in the work place according to PTSD criterion A4 of the DSM-5 and the Work and Social Adjustment Scale (WSAS) [21] for work and social functioning.

The TALS-SR was developed by the authors, who comprise an international (Italian–American) collaboration research project named Spectrum Project (http://www.spectrum-project.org/), established to develop and test assessment instruments for assessment of the spectrum of clinical features associated with the current version of the DSM psychiatric disorders. In particular, the TALS-SR includes 116 items exploring the lifetime experience of a range of losses and/or traumatic events and lifetime symptoms, behaviors, and personal characteristics that might represent manifestations and/or risk factors for the development of a stress response syndrome. The instrument is organized into nine domains including loss events (I); grief reactions (II); potentially traumatic events (III); reactions to losses or upsetting events (IV); re-experiencing (V); avoidance and numbing (VI); maladaptive coping (VII); arousal (VIII); and personal characteristics/risk factors (IX). The responses to the items are coded in a dichotomous way (yes/no) and domain scores are obtained by counting the number of positive answers. In the Italian version, test–retest/inter-rater reliability was excellent, with infraclass correlation coefficient values exceeding .90 for each of the domains [18, 19].

The Work and Social Adjustment Scale is a test used to evaluate and measure the work and social adjustment. It includes five items that assess the individual's ability to perform the activities of everyday life and how these are affected, in the week prior to the assessment. The first item investigates the work ability of the subject. The second item assesses the ability to cope with household chores, such as cleaning the house, looking after the children and shopping. The third item assesses private recreational activities carried out by the patient, such as reading, going to cinema and museum. The fourth and fifth items investigate the family interaction and relationship: in particular, the fourth item investigates the social activities carried out exclusively with people who are not part of the family, and includes activities such as parties, tours of pleasure, go clubbing or show up to sentimental appointments. The fifth item analyzes only the relations with family members with whom the person lives, and if any problem of the subject under examination has interfered with this type of relationship. Each of the 5 item is rated on a nine-point scale ranging from 0 (not at all) to 8 (severe interference), so that the total scores are between 0 and 40 reliability [20].

In accordance with the aims of the study, all participants were asked to report symptoms related to work-related trauma exposure, referring to their work activity at the emergency room of the Azienda Ospedaliero-Universitaria Pisana (AOUP). Due to the sample characteristics, the criterion A was considered satisfied. According to previous studies [21–29], symptomatological PTSD prevalence rates according to either the DSM-IV-TR and the DSM-5 criteria were assessed by means of a matching between the TALS-SR and the DSM PTSD symptoms. In particular, in the present study a symptomatological DSM-5 diagnosis of PTSD was assessed by using the following matching between DSM-5 symptoms criteria and TALS-SR items, a scheme already used in 2014 to assess gender differences in PTSD scores on a sample of 512 survivors to the L’Aquila earthquake [23]:

- Criterion B (*B*1 = 80; *B*2 = 77; *B*3 = 79; *B*4 = 78; *B*5 = 81);
- Criterion C (*C*1 = 86; *C*2 = 87 and/or 88 and/or 89);
- Criterion D (*D*1 = 90; *D*2 = 95; *D*3 = 85; *D*4 = 96; *D*5 = 91; *D*6 = 93; *D*7 = 92); and
- Criterion E (*E*1 = 108; *E*2 = 99 and/or 100 and/or 102 and/or 103 and/or 104; *E*3 = 106; *E*4 = 107; *E*5 = 105; *E*6 = 109).

### Statistical analysis

In order to compare the rates of PTSD in the groups examined, we adopted exact Fisher test. As considered variables are not normally distributed, we adopted non-parametric Mann–Whitney test to compare groups. In particular, the study protocol provided for a total sample of 120 subjects in order to ensure an 80% statistical power when comparing groups for the total WSAS scores, considering an effect of clinical relevance a difference of at least two points related to an expected standard deviation within groups equal to 5.

All the processing statistics were conducted using the Statistical Package for Social Science (SPSS Inc.), version 22.

## Results

A total sample of 42 subjects was included in the study: 32 (76.2%) nurses and 10 (23.8%) health care assistants. Among the whole sample, 13 (31.0%) subjects of the total sample were males, 22 (52.4%) aged below 40 years old, 25 (59.5%) graduated (this included only younger nurses that according to new Italian laws require a University Degree).

With regard to the presence of PTSD, nine subjects (21.4%) met DSM-5 criteria for the diagnosis of the disorder with higher, despite not significantly, rates among health care assistants, females, older, and non-graduated subjects (see Table 1).

**Table 1:** Prevalence of PTSD and comparison of WSAS total mean scores among study groups

	Total sample *n* (%)	PTSD		Total WSAS	
		Rates *n* (%)	(Fisher) *p*	Mean ± SD, (mean rank)	(Mann–Whitney) *p*
Occupation					
Nurses	32 (76.2)	6 (18.8)	.660	7.06 ± 6.94, (21.61)	.919
Health care assistants	10 (23.8)	3 (30.0)		8.10 ± 9.78, (21.15)	
Gender					
Males	13 (31.0)	2 (15.4)	.695	5.15 ± 7.42, (16.88)	.103
Females	29 (69.0)	7 (24.1)		8.28 ± 7.59, (23.57)	
Age					
≤ 40 years old	22 (52.4)	4 (18.2)	.714	6.91 ± 7.63, (20.70)	.658
> 40 years old	20 (47.6)	5 (25.0)		7.75 ± 7.72, (22.38)	
Education					
Graduated	25 (59.5)	5 (20.0)	1.00	6.80 ± 7.59, (20.30)	.440
Non-graduated	17 (40.5)	4 (23.5)		8.06 ± 7.75, (23.26)	
Total	42 (100)	9 (21.4)			

When comparing the TALS-SR symptomatological domain IV, that explores the acute reactions to losses or traumatic events, between subsamples, we found statistically significant higher scores in health care assistants, older, and non-graduated subjects (see Table 2).

**Table 2:** TALS-SR domain IV (reactions to losses or upsetting events) scores comparisons between subgroups

Groups	Reaction to losses or traumatic upsetting events	*p*
	Mean ± SD	
Occupation		
Nurses (*n *= 32)	4.50 ± 2.93	.028
Health care assistants (*n *= 10)	7.00 ± 3.33	
Age		
≤ 40 years old (*n *= 22)	4.09 ± 2.82	.030
> 40 years old (*n *= 20)	6.20 ± 3.24	
Education		
Non-graduated (*n *= 17)	6.65 ± 3.16	.007
Graduated (*n *= *25*)	4.04 ± 2.78	

## Discussion

The low percentages of response to self-assessment questionnaires are largely attributable to the fact that the design of the study provided that the questionnaires were fulfilled immediately before or after operators work schedule and many subjects declined and complained.

Nevertheless, the results of the present study corroborate increasing literature highlighting the fact that emergency services personnel face frequent exposure to potentially traumatic events with the potential for chronic symptomatic distress, such as PTSD and post-traumatic stress spectrum symptoms [14, 30–32].

According to previous literature [1, 3, 11, 16] we reported PTSD rates among health professionals operating in the emergency in Italy higher than those reported, despite with different methodological approaches, in the general population [6, 33]. The rates we found are in the range of prior works developed worldwide [3, 14, 34, 35], but appear to be in the higher range of prior works on nurses and health care workers operating in the emergency services across Europe, where PTSD prevalence rates ranging between 10 and 21% have been reported [8, 16, 34, 36]. Clohessy and Ehlers [8], in fact, exploring a sample of paramedics and emergency medical technicians in the UK, showed that 21% of enrolled subjects was affected by PTSD, diagnosed by both DSM-III and DSM-IV. Maia and Ribeiro [16], reported high exposure to events as evaluated as traumatic but low prevalence of PTSD (3.4%) among 59 between nurses and medical staff from the National Institute of Medical Emergency in the north of Portugal. Bennet and colleagues [36], exploring the psychological reactions in a sample of emergency ambulance personnel in a combination of rural and urban setting in the UK, found a 22% overall rate of PTSD. Further, Johnson and colleagues [34] showed a prevalence of post-traumatic stress disorder around 15% among Swedish ambulance personnel.

Our results also seem to be in line with the current literature which recognizes female gender as a risk factor for PTSD [37, 38]. However it is important to notice that studies exploring the possible confounding role of other risk factors for PTSD, including work-related training and education, reported gender difference may be lowered when a specific professional training has been performed, as reported among nurses [11], police officers, and fire workers [12, 39].

It is also noteworthy to underline as higher PTSD rates, as well as higher scores in all the symptomatological TALS-SR domains, were found among health care assistants with respect to nurses, as well as non-graduated with respect to graduated subjects. Some authors highlighted a possible relationship between education level and PTSD [13, 40]; accordingly, epidemiological data from the ESEMeD study in Italy reported significantly higher PTSD rates in subjects with lower instruction levels [6]. Despite conflicting results have been reported in the possible relationship between education level and PTSD [35, 39], the strongest evidence seem to suggest lower levels of education to render subjects at higher risk for PTSD. Health care assistants represent a professional category in Italy that perform basic care and administrative duties in a doctor’s office, clinic or hospital, and their specific roles vary by health care specialty, but common duties include gathering patient information, checking vital signs, taking notes during a physician’s visit. Upon Italian legislation, no bachelor’s degree is required for this role. For what concern nurses, Italian legislation changed since 1994, when a specific bachelor’s degree became a compulsory requirement to be hired by public and private health services. Thus, younger nurses all acquired a higher education level with respect to older ones. We may thus argue that our results suggest a preventive role of professional education on the risk to develop PTSD according to previous data [13, 16, 40]. In fact, Mealer and colleagues [11] highlight the possible development of resilient coping strategies among nurses during the course of their professional career. These considerations corroborate the importance of education and training as an important prevention factor in the developing of PTSD.

Our results show older subjects reporting higher rates of PTSD with respect to younger ones and we speculate that most likely older subjects reported the lower education levels, adding evidence to the previous findings. In addiction, some authors correlate the time spent in the same working place with an increased risk for PTSD and a more severe symptomatology [4, 41]. Specifically Berger and colleagues [4] reported higher incidence of PTSD in specific occupational groups of rescue workers including those with a longer job experience.

Interestingly, we reported statistically significant higher post-traumatic stress spectrum scores in the TALS-SR domain exploring symptoms of acute reaction to trauma. Prior studies documented the relationship between acute distress reaction and the risk of PTSD highlighting the fact PTSD represents the ideal candidate for secondary prevention programs [42, 43]. In this regards, Maia and Ribeiro [16] found peri-traumatic dissociation and distress symptoms to be the only predictor of PTSD symptoms among a group of health emergency operators.

The finding regarding the severity of impairment in work and social-related adjustment in the randomized groups strengthens the consideration that women with a lower educational level are more at risk to develop work trauma-related PTSD symptomatology.

Interpretation of our results should keep in mind some important limitations of the study. As already mentioned, the most important is related to the limited sample size and the lack of a control group of health worker not employed in the Emergency Department that could affect the methodological strength of our study and consequently the generalizability of our results; nevertheless, this is a pilot study with preliminary data on a topic ignored in Italy in the actual literature which shed light on the need to better explore post-traumatic stress symptomatology on emergency health workers. Second limitation is the use of a self-report instrument to detect PTSD symptoms and even the diagnosis. We opted for an accurate self-report since the only comparable international validated instrument for PTSD is the Clinician-Administered PTSD Scale (CAPS) which is a semi-structured interview and we dreaded for high percentages of drop out and risk of bias of untruthful answers as we were on the workplace of the subjects recruited. A further limitation of the study is the lack of information on the presence of Axis I psychiatric comorbidities that may have an impact on some of the variables explored, particularly the work and social functioning levels.

## Conclusions

Despite the limitations mentioned above, this report suggests emergency health professionals, in particular older subjects and women, to be a category at high risk of post-traumatic stress spectrum. Further research is thus needed to advance scientific understanding on the real impact of trauma on this population in order to better clarify which are the categories at higher risk and develop specific education and training interventions for prevention programs.
